# The Story of the Insane from Year to Year

**Published:** 1899-08-12

**Authors:** 


### THE STORY OF THE INSANE FROM YEAR TO YEAR.

(Eleventh Series).

### WEST SUSSEX ASYLUM AT CHICHESTER.

This is the first annual report, and it contains ground and block plans of the asylum. From these we see that the building is something between the "horseshoe" and "broad arrow" plan, and a good deal may be said in favour of this arrangement; but some means should have been devised of placing the workshop and laundry wards outside of the corridors, thereby obviating the cheerless view of inner-court walls. The isolation hospital has two dormitories for three beds each, a number which would be hopelessly insufficient in the event of an epidemic spreading. We notice also that the bath-room and closets for this hospital are placed off the main corridor, instead of being attached to the dormitories by ventilating passages. On the other hand, many of the rooms in the main building must be light, cheerful, and airy. There were 441 admissions, 2 discharged recovered, and 6 deaths. On the last day of the year there were 430 patients in residence, and as the asylum is intended to accommodate 450, it is absolutely certain that building operations will soon have to be commenced. Why the asylum was not made of a larger size to begin with is a mystery; and yet it is one of the commonest mistakes, although the annual increase in the number of the insane is a fairly well-known quantity. The committee record "their appreciation of the able way in which Dr. Kidd has discharged his onerous duties as medical superintendent." As in all newly-opened asylums, the weekly rate of maintenance is high.

### THE HEREFORD COUNTY ASYLUM.

This is one of our smallest county asylums, containing on the last day of December, 1897, only 360 patients. There were 50 first admisions, 23 readmissions, 19 recoveries, and 23 deaths. Calculated on the year's admissions the recoveries stand at 26 per cent., and the deaths at 6·1 on the average number resident. Both of these percentages are low; that of the deaths decidedly so. Fifteen of the deaths were caused by cerebral and spinal diseases, and only 2 by consumption. Moral causes account for 17 of the year's admissions, and drink for 8, while hereditary tendency is credited with being the cause in 29 instances. The asylum is full, and contracts have been entered into with the East Riding Asylum and with Grove Hall Asylum to receive a total af 50 patients. Additions have been sanctioned for 150 patients at an estimated cost of £52,000. Apart from the crowded state of some of the wards and the transfer of chronic cases to other asylums, the institution seems to have been free from any disturbing element during the year. The visitors say that Dr. Morrison "has shown a great interest and been helpful in working out the various details connected with the proposed enlargement of the asylum." The weekly cost of maintenance is a little over 9s. 1d. per head.

